# SEND: a system for electronic notification and documentation of vital sign observations

**DOI:** 10.1186/s12911-015-0186-y

**Published:** 2015-08-13

**Authors:** David Wong, Timothy Bonnici, Julia Knight, Lauren Morgan, Paul Coombes, Peter Watkinson

**Affiliations:** 1 Institute of Biomedical Engineering, Old Road Campus Research Building, University of Oxford, Oxford, OX3 7DQ UK; 2 Kadoorie Centre for Critical Care Research and Education, Level 3, John Radcliffe Hospital, Headley Way, Oxford, OX3 9DU UK; 3 Botnar Research Centre, Nuffield Orthopaedic Centre, Windmill Road, Oxford, OX3 7LD UK; 4 IM&T, John Radcliffe Hospital, Headley Way, Oxford, OX3 9DU UK

**Keywords:** Early warning, Electronic, Track and trigger, Vital signs

## Abstract

**Background:**

Recognising the limitations of a paper-based approach to documenting vital sign observations and responding to national clinical guidelines, we have explored the use of an electronic solution that could improve the quality and safety of patient care. We have developed a system for recording vital sign observations at the bedside, automatically calculating an Early Warning Score, and saving data such that it is accessible to all relevant clinicians within a hospital trust. We have studied current clinical practice of using paper observation charts, and attempted to streamline the process. We describe our user-focussed design process, and present the key design decisions prior to describing the system in greater detail.

**Results:**

The system has been deployed in three pilot clinical areas over a period of 9 months. During this time, vital sign observations were recorded electronically using our system. Analysis of the number of observations recorded (21,316 observations) and the number of active users (111 users) confirmed that the system is being used for routine clinical observations. Feedback from clinical end-users was collected to assess user acceptance of the system. This resulted in a System Usability Scale score of 77.8, indicating high user acceptability.

**Conclusions:**

Our system has been successfully piloted, and is in the process of full implementation throughout adult inpatient clinical areas in the Oxford University Hospitals. Whilst our results demonstrate qualitative acceptance of the system, its quantitative effect on clinical care is yet to be evaluated.

**Electronic supplementary material:**

The online version of this article (doi:10.1186/s12911-015-0186-y) contains supplementary material, which is available to authorized users.

## Introduction

In the UK alone, there are estimated to be over 10,000 avoidable in-hospital deaths each year [1]. Avoidable mortality may be reduced through improved recognition of abnormal vital signs, which are known to be correlated with adverse events such as unexpected cardiac arrest [2].

To facilitate the documentation and recognition of abnormal vital signs, we have designed and developed bespoke software, System for Electronic Notification and Documentation (SEND). SEND allows vital signs to be entered and viewed. It also provides basic clinical decision support based upon our hospital’s Early Warning Score protocol.

## Background

The standard of care in UK hospitals is routine monitoring of the basic vital signs–heart rate, respiratory rate, blood pressure, oxygen saturation, temperature and consciousness level–at least every 12 h with the values of the vital signs being input into an Early Warning Scoring (EWS) algorithm [3]. An EWS is calculated by assigning integer (weight) to each vital sign according to a lookup table, and then summing the scores. The score is used to determine how regularly future vital sign observations should be recorded. Documentation in the majority of hospitals is carried out using paper-based vital signs charts with manual calculation of the EWS.

This system has a number of flaws. Errors are frequently made in calculating EWS, either due to incorrect assignment of weights or errors in summing them. The error rate may be high. In one study, 20 % of documented observations recording in an Emergency Department setting had an incorrect EWS [4]. Other criticisms of paper-based EWS systems include poor legibility of clinical notes, and a difficulty in physically accessing these notes [5].

The NICE CG50 recommendations highlighted electronic monitoring systems as a potential method to identify patients at risk of clinical deterioration [3]. More recently, a review of current practice in the Mid-Staffordshire NHS Foundation Trust also recommended that: “vital signs should, where possible, be done automatically as they are taken, with results being immediately accessible to all staff electronically in a form enabling progress to be monitored and interpreted”. (Recommendation 243) [6]. Over the last decade, both standalone systems [7–9] and charting modules for Electronic Patient Records [10] have been developed and used within acute hospital settings.

The introduction of these electronic systems has been linked to improvements in accuracy and timeliness of observations [11]. Prytherch et al. has also reported that electronic assistance may reduce the time required to document observations. Within a classroom environment, electronic documentation of a set of vital sign observations was completed 1.6 times faster than paper documentation [12]. The overall clinical benefit (i.e. length of stay, patient mortality) of electronic EWS systems is still an active research subject and recent studies have produced conflicting outcomes, with Schmidt et al. noting a reduction in mortality after the introduction of such a system, whereas Dawes et al. showed no such change using the same system [13, 14].

### Motivation

We initially sought to develop a set of key functional requirements for the SEND electronic vital sign documentation system. Existing paper charts have a number of features which are desirable to preserve: portability, resilience to failure, and, for medically-trained personnel, the low cognitive load imposed by entering and reading data. Our system specification was governed by two over-riding principles. First, that the system should enable best practice to be achieved more easily than on paper; and second, that the system should maintain all the benefits of a paper system. The requirements were formalised using the process defined in IEEE standard 1233-1966 [15]. Four clinicians, two doctors and two nurses were embedded within the team and acted in the “customer” role during the specifications process.

Initial “raw” requirements were specified using three sources of information, clinical expertise, published literature on nursing practice of recording vital sign observations (using both paper and electronic charts), and a formal ethnography study of the practice of recording vital signs on Level 1 [16] wards across multiple specialties. The prototype was refined using two processes, a formal Failure Modes and Effects Analysis carried out by two trained ergonomists, and a User Centred Design process whereby nursing staff unrelated to the team were observed using the prototype. A number of requirements were critical in determining the technical approach to building such a system. They are listed in Table 1, and the key software and hardware design decisions are described in more detail in the remainder of this article. We then present a brief description of the SEND system, and report results on its uptake within the Oxford University Hospitals.

**Table 1:** Specifications for an electronic vital sign documentation system developed following analysis of nursing practice

Specification	Derived requirement	Implementation
Reliable and accurate user and patient identification with minimal reliance on supporting systems and infrastructure	Identification of the patient using demographics encoded within the PDF417 barcode on the patient’s ID wristband	*Hardware*: 2D barcode scanner
		*Software*: Allow patient identification through 1D or 2D barcode wristbands
Minimise the time to enter and access data	• Minimise time for device wake/login	*Hardware*: capable of running one of the mobile operating systems (iOS, Android, Windows 8)
	• Real-time data entry validation	*Software*: Minimise requests to server by building highly-dynamic web pages, and completing rudimentary data processing on the client
Encourage contemporaneous data entry at the bedside by minimising physical workload or the possibility of items needed to complete the task being unavailable [19, 20].	Ensure that all equipment required for the task is co-located	*Hardware*: Mount the screen and barcode scanner on the same stand as the monitoring equipment
Support users in applying their clinical judgement by minimising the physical and cognitive workload. Attempt to avoid the unintended consequence of users supressing their own judgement in favour of the interpretation provided by the system [21].	• Staff should be able to see previous vital signs at the time of data entry to facilitate interpretation	*Hardware*: Sufficiently large screen to enable a chart view that is easy to interpret.
	• All vital signs in an observation set should be visible without requiring the user to scroll	
	• Data should be readable by users who may not have perfect visual acuity.	
	• Wherever possible, data should be presented graphically [22]	
The physical and mental workload to review data should be minimised	• Data should be viewable on computing devices used for clinical data access within the Oxford University Hospitals Trust as well as accessible within the Oxford University Hospitals Trust’s Electronic Patient Record (Cerner)	*Software*: Web-based application that works on all platforms and can be embedded within third part systems.
	• The data should be displayed in the same format wherever possible	
All hardware and software must Adhere to the local hospital trust policies		*Hardware*: Must comply with infection control, and health and safety regulations
		*Software*: Data must be stored securely

## Implementation

### Software

The SEND software was built as a web-based application to provide us with greater flexibility for deployment across multiple hardware platforms (see Table 1). The technology stack chosen for the application was a MySQL database, PHP client-side language (using the CakePHP framework), and HTML, CSS and Javascript. The Javascript data-binding framework, Knockout.js, was used in addition to more standard libraries such as JQuery to enable highly interactive web views that closely mimic a native application experience. These web views allow vital sign observation data to be pre-processed within the web browser, so that the number of requests made to the server is minimised. This approach reduces the time required to enter vital sign observations (thereby meeting the initial criteria) by minimising the number of web page loads.

**Fig. 1:**
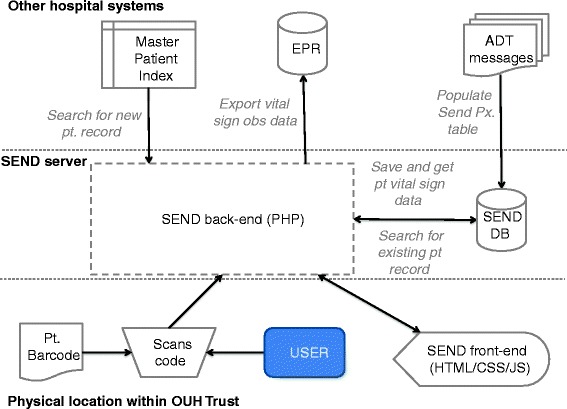
Data flow pathways for SEND. Data flow pathways for SEND, showing flow of patient data between the user, third party hospital systems and the SEND system

A key priority of the software design was to support reliable and accurate patient identification with minimal reliance on supporting systems and infrastructure. To design an appropriate solution, we first mapped the expected flow of information between users, SEND, and supporting systems (Fig. 1). The figure shows that SEND relies on near real-time Admission, Transfer, and Discharge (ADT) messaging to generate a list of active patient records, and a Master Patient Index that contains demographics for all patients who have ever been admitted to the hospital trust. These external sources of data are required to identify the patient.

Prior to any clinical task, clinicians are required to positively identify the individual using the patient’s name, date of birth, gender, hospital record number and National Health Service (NHS) number. To enable this, each patient admitted to the hospital is required to wear a wristband that displays these key identifiers. In addition displaying identifiers in plain text, they are encoded in two barcodes. All patient identifiers on the wristband are encoded within a PDF417 (2D) barcode and additionally the hospital record number alone is encoded as a 1D barcode for the benefit of legacy systems.

SEND allows a patient to be identified via either barcode. The 2D barcode is the primary method of identification because it allows a patient to be identified by their NHS number, as per NHS England stipulations. However, recognising that the PDF417 symbology was less familiar to users than the 1D barcode we implemented all fall back solution so that whichever barcode the user scans the patient will be identified.

For the 1D case, the barcode is decoded into the hospital record number, which is used to search the SEND database. The SEND database is updated in near real-time by the hospital’s ADT messages. If a patient record cannot be not found, SEND attempts to find the required patient demographics (Name, Date of Birth) from the Master Patient Index (MPI). If this is successful, a temporary patient record may be added to the SEND database. The temporary record is automatically merged with a matching active record once the relevant ADT messages have been processed and the SEND database has been updated.

For the 2D case, the barcode is decoded into the patient demographic information described previously. Like the 1D case, the SEND database is then searched for a matching patient record; if no match is found, a temporary record is created if the relevant data from the MPI can be retrived.

The 2D barcode has one further advantage. In the fringe case where it is necessary to record observations before a complete electronic patient record is available, a temporary record may be created using data in the 2D barcode, without reference to any external data sources. This provides robustness by reducing reliance on inter-system communication, meeting our initial specification.

The SEND web-application was developed to adhere to the Oxford University Hospitals’ data protection policies. Control of patient-sensitive information is ensured by storing all patient-identifiable data on the locally-hosted server. Access to the SEND application is only granted to devices connected to the hospital intranet via HTTPS. Furthermore, client devices are limited to only allow access to one patient record at a time. By employing trust policies on data caching within browsers, the risk of compromising patient data is minimised for the event that a client device is stolen.

### Hardware

**Fig. 2:**
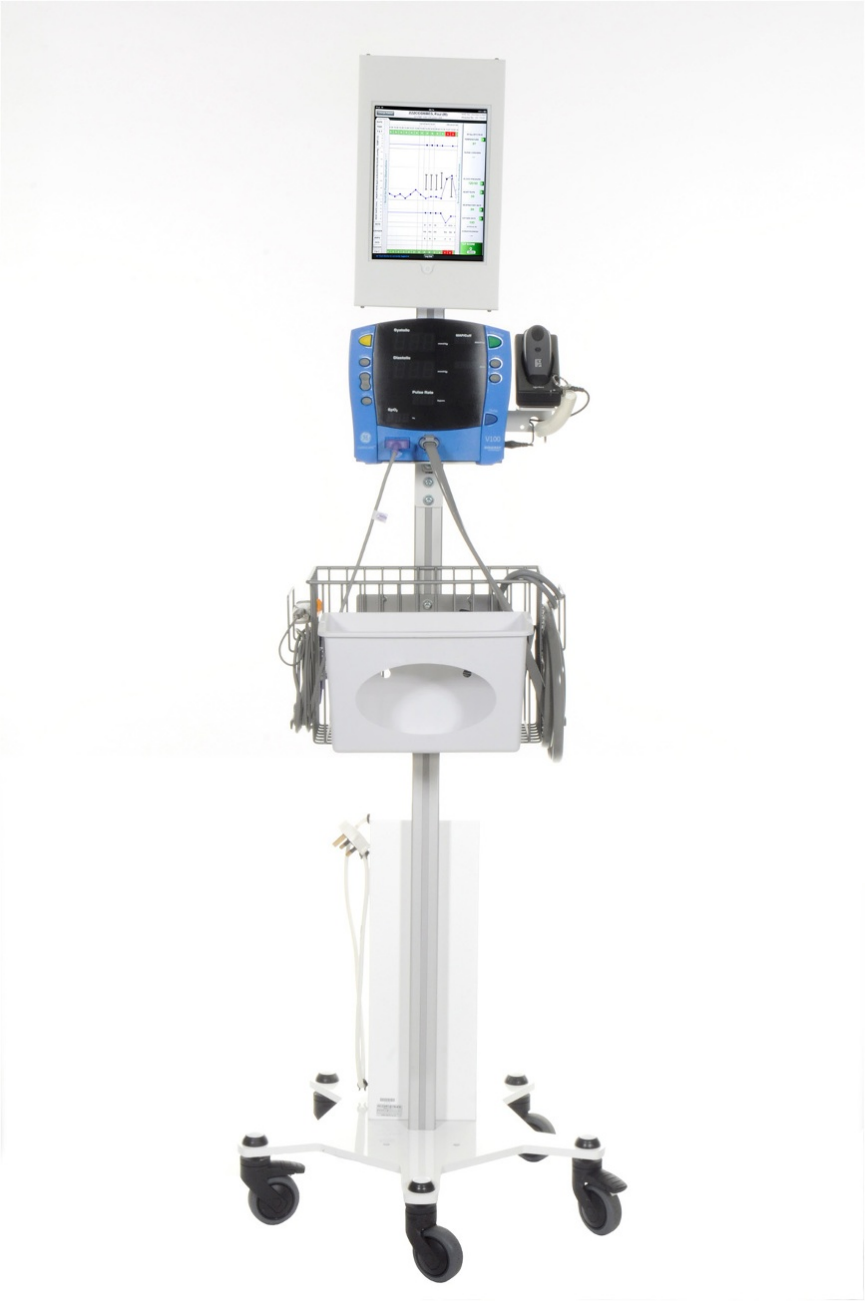
The prototype SEND stand. The two novel design features of the roll stand were the mounting for the tablet computer and the provision of an enclosed power distribution board to which both the patient monitor and tablet computer power cables were connected. This enables all the system components to be charged using a single cable

Our pre-development work suggested that co-location of the vital signs monitor with the system for vital sign recording and display would optimise process reliability and efficiency. Through the development and manufacture of a bespoke stand, the hardware setup was designed to ensure that all equipment required for vital sign observation was integrated into a single mobile unit. The stand accommodates the data-input device, barcode scanner and monitoring equipment. The stand provides a flexible mounting system to accommodate different models of vital sign monitor. The stand is depicted in (Fig. 2) and notable features are highlighted. This setup is unique to SEND; in other electronic vital sign recording systems, data-input devices are often charged and stored separately from the vital sign monitoring equipment. This is by necessity, as monitoring equipment is typically attached to stands without capacity to charge the data input devices.

#### Barcode scanner

The patient identifiers are encoded on the patient wristband using the PDF417 symbology, which consists of stacked linear barcodes. The vast majority of linear barcode scanners (1D scanners) cannot decode PDF417 data. Therefore we specified that an area imager (2D barcode scanner) should be used for the SEND system. 2D scanners have the additional advantage of avoiding being able to decode barcodes irrespective of the pitch and yaw of the barcode relative to the scanner.

Three types of 2D barcode scanner were considered: wired scanners interfacing via USB cable (or USB-to-lightning cable for iPads), wireless scanners interfacing via Bluetooth and software barcode readers which use a tablet’s integrated camera to image the barcode. 2D Bluetooth barcode scanners from 4 different manufactures and 8 software barcode scanning libraries were assessed.

Interfacing a 2D scanner with an iPad was technically impossible. The peak current draw of the most efficient wired 2D scanner we could find (the Motorola DS4208) exceeded the iPad’s current limit of 100 mA. Using a camera or scanner integrated into a fixed, pole-mounted tablet would have been ergonomically unacceptable so this option was discounted.

The use of a wired barcode scanner is limiting. Few tablets provide both a USB port and a separate power port. Wireless barcode scanners have the advantage of allowing any modern tablet computer to be used. However, system reliability analysis highlighted that the use of wireless scanners would greatly decrease reliability due to depletion of the scanner battery, failure of Bluetooth pairing or data transmission and removal of the scanner from the stand (unless it was tethered by a cable to the stand). Testing of the candidate Bluetooth scanners confirmed that these failures occurred commonly.

An additional disadvantage of the wireless scanners was that the scanning engines in the wireless scanners we evaluated appeared to be slower and be more affected by barcode imperfections and resolution than the wired Motorola DS4208 scanner. Furthermore, the cost of the wireless scanners was significantly higher than the wired scanners. Therefore a decision was taken to use wired 2D barcode scanners (Motorola DS4208), interfacing using USB.

#### Tablet

The choice of tablet was limited by two factors. Firstly, the tablet needed to support a modern web browser to allow full functionality of the SEND web application. Secondly, we needed to be able to charge the tablet whilst a wired USB barcode scanner was connected.

Android devices were tested and discounted due to limitations of the USB-on-the-go protocol. iOS devices were similarly discounted, as no Lightning- to-USB adaptor currently exists which allows simultaneous charging and USB data transfer. Therefore, our system uses a tablet running the Windows 8 operating system. This has the further advantage of being familiar to the hospital’s technical support team.

### Monitoring usage and usability

We assessed SEND, following its implementation, to determine whether it enables vital signs to be documented as expected, and whether the system is acceptable to users. We undertook this assessment using two methods. Firstly, we calculated the number of active users and the number of observations taken using SEND. We defined an active user as anyone that has used SEND to record a set of vital sign observations within the previous 14 days. These metrics were compared to lower-bounds that were calculated as follows:

- Number of active users–active users should the figure should correspond to the nursing staff levels obtained from hospital ward audit data.
- Number of observations–We assume that wards are always at capacity and that each patient is observed at least once every 12 h, in keeping with national guidelines. Therefore, the minimum expected number of observations in 1 week is given by:

$$ \mathrm{number}\ \mathrm{of}\ \mathrm{observations}\ \mathrm{per}\ \mathrm{week}=\mathrm{active}\ \mathrm{beds}\times 2\times 7. $$

Secondly, to obtain an overall indication of the level of usability of the system, all new system users (including doctors, nurses, allied healthcare professionals and students) were asked to complete a feedback form approximately 2 weeks after SEND had gone live in the clinical area. The form contained a ten question system usability score (SUS) section that was based on a Likert scale [17]. The responses to each question were graded from strongly agree to strongly disagree; the full list of these questions is given in Additional file 1.

## Results

### SEND

The initial version of the SEND system was completed and first introduced into clinical practice in March, 2014. We now present an overview of SEND’s two primary functions: recording vital signs, and reviewing a patient.

#### Record vital signs

**Fig. 3:**
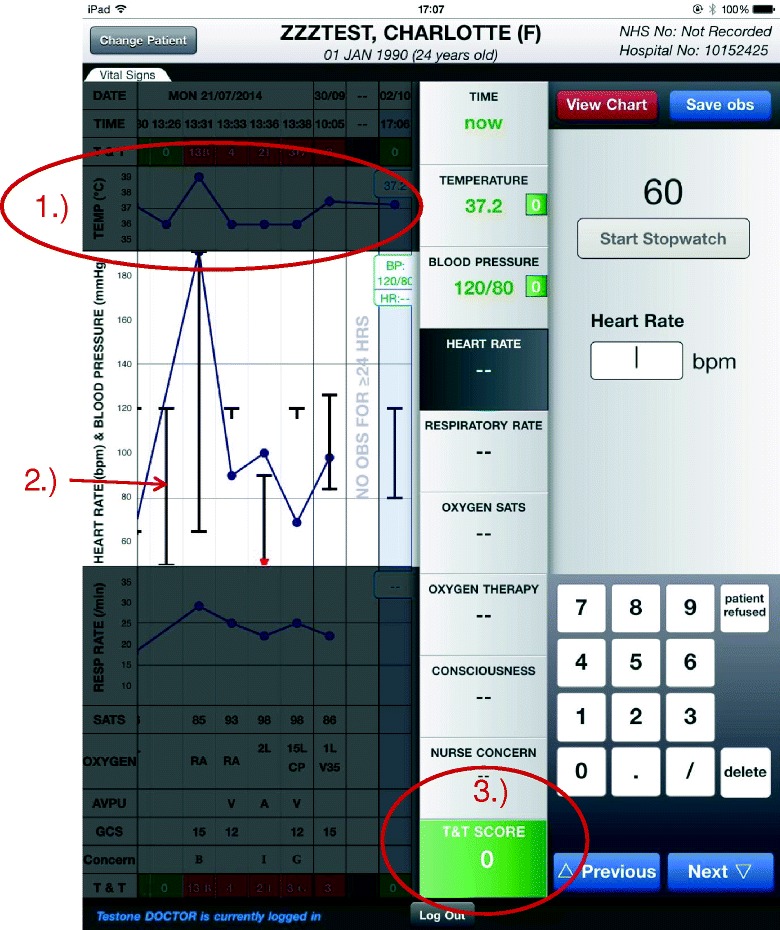
The ‘Record Vital Signs’ view. Three key features of this view are highlighted in *red*: *1*.) Historical vital sign values are visible and charted in a manner that is similar to paper observation charts, *2*.) The trend for the current field (HR) is *highlighted*, and irrelevant chart areas are *greyed*-*out* to reduce cognitive load, *3*.) EWS sub-totals and total scores are calculated in real time and displayed prominently

The *record vital signs* screen allows users to record a new set of vital signs for a patient. Access to the *record vital signs* screen occurs when a SEND user scans their identification badge barcode on the initial login page on a dedicated ‘data capture’ device. The user is then redirected and asked to scan the 2D barcode on the patient’s identification wristband. Upon successful identification of the user and patient, SEND displays a patient observation chart view and can enter observations immediately, as shown in Fig. 3. This process positively identifies both the patient and caregiver, and is designed to minimise the probability of observations being saved to the wrong patient record.

As Fig. 3-1 shows, previous observation sets are visible when entering the new vital signs, and the currently selected vital sign is highlighted to focus the user’s attention. As the user enters vital signs using the on-screen keyboard, the EWS score is calculated and displayed in real-time Fig. 3-3. The visual feedback is designed to minimise EWS calculation errors, and to provide immediate feedback to the user as to which vital sign is abnormal.

The observation data are saved to a dedicated SEND server, and are simultaneously exported, via HL7 messages, to the hospital Trust’s Electronic Patient Record. Once the observations have been saved, the user is provided with advice relevant to the current EWS score. The design of the observation chart closely matches that of the hospital trust’s paper equivalent. In particular, blood pressure measurements are plotted using the standard medical notation, with lines indicating Systolic and Diastolic Blood Pressure levels and a single connecting line between the two Fig. 3-2.

#### Patient review

**Fig. 4:**
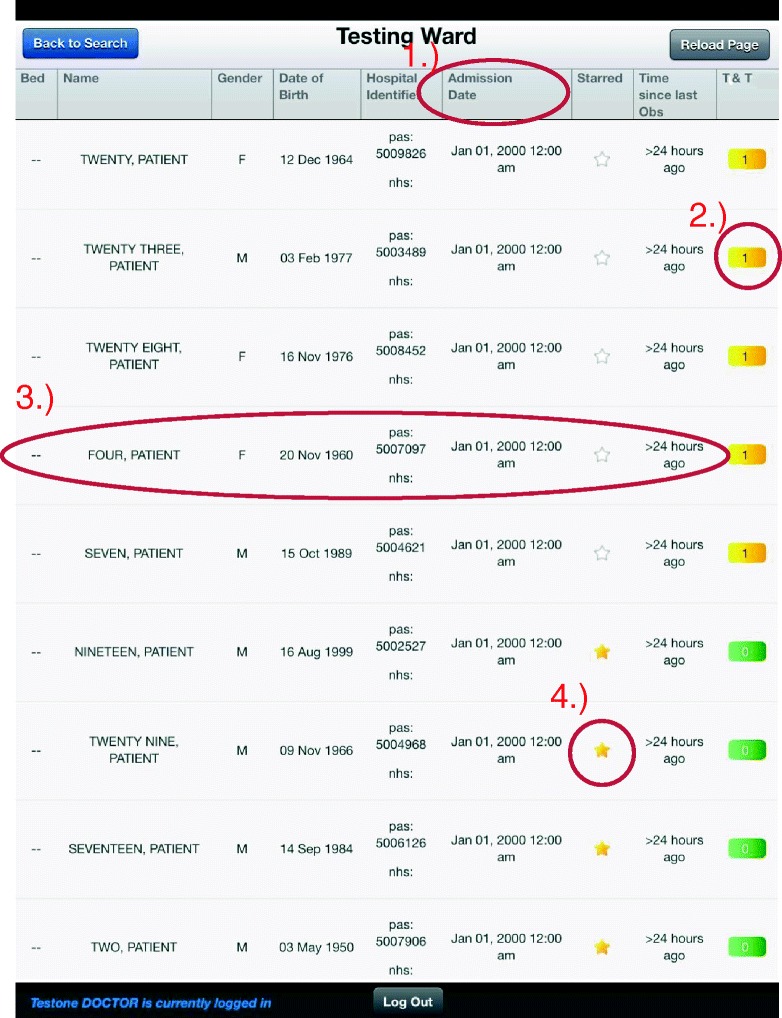
The ‘Patient Review’ view. A typical ward list, showing all patients on a ‘Testing Ward’. Four features are *highlighted*: *1*.) Each table heading may be selected to enable bespoke sorting, *2*.) Selecting the EWS score brings up a panel showing the latest set of vital signs, *3*.) Selecting the patient’s row redirects the user to the observation chart, *4*.) Each patient can be ‘starred’ and saved to a user-specific list

The *patient review* screen allows nurses to easily view all patients currently admitted to a ward on one screen (Fig. 4) and to access their observation charts. We envision that the review screen will be useful during nurse handover and doctors’ ward rounds, in which the ability to quickly switch between multiple observation charts is paramount. Accessing the patient review screen requires only two user actions. Firstly, the SEND user logs in on a ‘review’ device by scanning or typing their identification badge. The identification badge is not required to be unique to SEND; within our hospital trust, pre-existing staff badges were used. The user is then presented with a searchable list of hospital wards. When a ward is selected, the ward review screen is displayed. The patient list is continuously updated using admission, discharge and transfer (ADT) messages collected from the hospital’s electronic patient administration system. The patient list is initially sorted in descending order by EWS score, so that patients with more physiologically abnormal vital signs are immediately recognised. The list may also be sorted by selecting any column heading, and secondary sorting is also supported Fig. 4-1. Such sorting may be useful, for instance, when a senior nurse may want to quickly determine whether any patients on the ward have been left unattended for an extended period.

Additional features of the review screen are highlighted in Fig. 4. Figure 4-2 shows that the current EWS score is displayed for the patient. When the score is selected, the EWS score expands to show the latest vital signs and EWS weights for the patient. This may be useful for determining the cause of an elevated EWS score. A patient’s full observation chart may be accessed by selecting the patient row Fig. 4-3. The final highlighted feature Fig. 4-4 shows the ‘starred’ patient system, in which patients may be selected on a per-user basis and saved to a separate list. In practice, this is useful for clinicians who may be caring for patients across multiple clinical areas.

#### System uptake

**Fig. 5:**
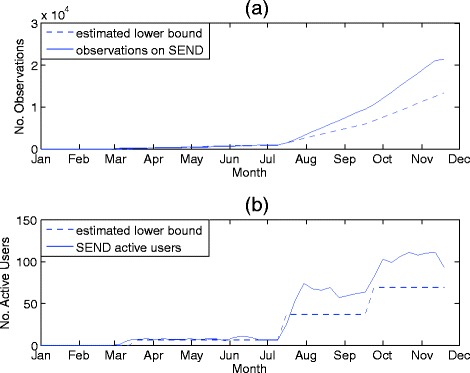
Uptake of the SEND system in clinical practice. **a** shows the number of observations recorded using the SEND system between March and December 2014. The total number of observations (*solid*) exceeds the minimum expected number of observations (*dashed*). **b** shows the number of active SEND users (i.e. those who have taken an observation set within the previous 14 days). The two large step changes correspond to SEND being implemented on a new ward. The number of active users (*solid*) exceeds the minimum expected number of active users (*dashed*), as determined by an audit of ward staffing levels

The SEND system has been deployed on three wards within the Oxford University Hospitals: a short-stay unit, an Oncology ward, and a respiratory ward. These wards contain a total of 59 beds. The system has completely replaced paper-based observation charting and has been in normal clinical use for 9 months at the time of writing. Data from the SEND database is summarised in Fig. 5.

Figure 5a shows the cumulative number of observations since the introduction of SEND in March, 2014. As of November 2014, 21,316 observation sets have been recorded on the system. This figure exceeds the lower bound on the number of observations (plotted as a dashed line), which was calculated according to the method described previously. As expected, we also see an increase in the rate of electronically-documented observations when new wards began using SEND in July 2014 and September 2014. Figure 5b shows the number of active users over time in comparison to a lower-bound of expected number of users (plotted as a dashed line); at the time of writing SEND has 111 active users. The lower-bound is derived from information on nursing staff numbers for each ward and does not take into account temporary staff numbers. As expected, the number of active users increases over time, with step changes when new wards transition from the paper-based charting approach to SEND. Between the addition of new wards onto SEND, the number of active users remains fairly constant, and fluctuations may be attributed to changes in the number of temporary staff from week-to-week.

The feedback form described previously was distributed to all new users 2 weeks after the initial implementation of SEND to the pilot wards. Users were able to complete the feedback form electronically or via a paper copy, and emails with links to the feedback form were sent to the pilot clinical areas on two occasions. This led to 65 completed feedback forms being received, of which the majority were submitted via the electronic form.

Using the pre-defined scoring methodology [17] applied to the ten questions listed in Additional file 1, the overall SUS was calculated as 77.8; all scores above 68 may be considered as ‘above average’. The SUS may be normalised to give a percentile rank. In comparison to over 3400 System Usability Scores collated in other settings, this rank falls within the upper quartile and is classified as ‘Good’ on a relative adjective rating scale [18].

In addition to the questions listed in Additional file 1, further questions regarding the system implementation were posed to assess whether clinical users perceived the level of training on SEND to have been adequate. When users were asked if they had been ‘…shown how to use SEND’ (input patients’ observations/ review patients’ charts) 95.3 % (62 of 65) replied ‘Yes’. Of those who had been shown how to use the system, the level of training was assessed as ‘not enough’, ‘too much’ or ‘just right’ by 4.8 % (3 of 62), 0 % (0 of 62) 95.2 % (59 of 62) of participants respectively.

## Discussion

SEND is not the first electronic vital signs charting system to be developed. However, its architecture is relatively unusual. Architectures of other widely available systems are:

1. Data entry via an electronic patient record using a computer mounted on a mobile cart [19].
2. Integration of Wi-Fi and barcode scanning into the monitor itself [23, 24].
3. Retro-fitting a monitor with a data entry and transmission module [25].
4. Handheld device (e.g. iPod Touch) for data entry and tablet or desktop computer for data review [26].
5. Use of small tablets (e.g. with 8″ screens) for data entry [27].

Data entry using a computer on a mobile cart on a general ward has been shown to be slower than use of a tablet mounted on the same stand as the monitor [19] and increases the rate of retrospective transcription [20]. Manipulating both a computer cart and a vital signs monitor into a bed space may be difficult, especially when the curtains are drawn around the bed to maintain patients’ privacy. We judged this to only be a viable solution in higher dependency areas where there are dedicated computers and monitors at every bed space.

Use of monitors with in-built Wi-Fi and barcode scanners or retro-fitting of existing monitors with these capabilities is an attractive solution from a system reliability point of view. However, it is very expensive to replace all ward monitors with models that integrate Wi-Fi and barcode scanners. The retro-fitting model is also costly. Leaving aside the cost of the data entry/transmission module, a significant number of our institution’s monitors were not capable of being upgraded to transfer data wirelessly so would have had to be replaced.

Systems consisting of a hand-held device for data entry and a tablet or laptop for data review have the significant disadvantage of separating the tasks of data entry and data review, a problem shared by monitors with in-built Wi-Fi or retro-fitted monitors. At the time of data entry the clinician is required to respond to the new vital signs. Good decision making is aided by seeing the vital signs in context of the previous values. A blood pressure which is normal for the population may be abnormal for an individual who is chronically hypertensive. Similarly an oxygen saturation which is low for the population may be normal in an individual who has chronic respiratory disease. Separating the tasks of data entry and review places a barrier to staff practising in an optimal manner.

Hand-held devices and monitors are capable of displaying graphical charts but in our experience the charts are small and hard to read, especially for staff who have visual impairment. More commonly these systems display data in a tabular format, making it hard to spot trends quickly.

The significant advantage of the hand-held device model is that the device can be carried by the staff member at all times and used for multiple tasks, if the hospital invests in a fully digital electronic patient record with a mobile devices interface. This is a benefit shared by small tablets. The SEND tablet is only useful for data entry in situations where the monitor is as the bedside.

Small tablets, especially those with high resolution displays, have the additional advantage over the hand-held devices of having a screen which is large enough to display a graphical chart at a usable size. However, the use of untethered mobile devices brings an additional cost associated with device management, security and charging facilities. Rather than keeping the devices in the hospital some institutions have opted for a Bring Your Own Device (BYOD) model or allocating a tablet to each staff member.

For a single application, such as vital signs charting, the SEND architecture is cheaper than providing a tablet per staff member or multiple tablets per ward with additional security infrastructure.. However, if the tablets are used for multiple clinical tasks the cost-benefit ratio moves in favour or an individual tablet per staff member.

Should our institution transition to mobile devices as the primary method of accessing clinical data the SEND system can be quickly adapted to work efficiently. In such a case we would envisage using the tablet camera as the method of scanning barcodes.

In its current form the SEND system seems to be well accepted by staff. The SUS score is high, with mean scores for individual statements ranging from 3.9/5 to 4.3/5. Examining individual question, the lowest score was given in response to: “I find that the various functions within SEND are well integrated”. The highest scoring statement was “I think I would like to use SEND frequently”.

## Conclusion

We have designed, developed and deployed a system for the electronic documentation of vital sign data. Our design philosophy focused on minimising disruption to current working practice. By applying this philosophy to the hardware and software design, we developed a system that uses technology to streamline existing tasks. The solution we have designed and implemented is a standalone system but it is fully interoperable with existing technologies within the hospital environment, such as its Electronic Patient Record and Identification Badge systems.

Early analysis of the data recorded during SEND deployment indicates that the number of active users and observations recorded is consistent with estimates based on prior audit information. Furthermore, a system usability questionnaire shows a positive view of the system from clinical users. We are using feedback from the questionnaire to iterate and make further improvements to the system.

As the system is deployed through 62 further wards in the Oxford University Hospitals, we intend to quantitatively evaluate the system’s effect in two ways. Firstly, we will determine the impact of SEND on the time to record and document observations through a time-and-motion study. In this study, nursing tasks will be timed before-and-after the introduction of SEND. Secondly, we aim to evaluate whether at-risk patients are monitored more appropriately after the introduction of SEND by evaluating the promptness of intervention following a high EWS score in a trust-wide stepped-wedge design study. These two investigations aim to definitively answer the most pertinent questions concerning electronic EWS systems; firstly, whether such systems can be used to optimise nursing workflows, and secondly, whether such systems have a measurable impact on patient outcomes.

## Availability and requirements

**Project Name:** SEND (System for Electronic Notification and Documentation)

**Project Home Page:**

**Operating System(s):** Platform independent

**Programming language:** PHP, HTML, CSS and Javascript, MySQL

**Other requirements:** Modern web browser

**License:** AGPL

**Any restriction to use by non**-**academics:** license needed

## Additional file

Additional file 1: **Supplementary information detailing the questions and method used to assess the system usability score.** (DOCX 14 kb)
