# Supplementary material for: SEND: a system for electronic notification and documentation of vital sign observations
Source: BMC Med Inform Decis Mak. 2015 Aug 13;15:68. doi: 10.1186/s12911-015-0186-y (PMC4542116; doi:10.1186/s12911-015-0186-y)
Supplement: Additional file 1: — Supplementary information detailing the questions and method used to assess the system usability score. (DOCX 14 kb) [file 12911_2015_186_MOESM1_ESM.docx]

Supplementary information regarding the system usability scale score of SEND

The following questions were used to calculate a system usability score:

1. I think I would like to use SEND frequently
2. I find SEND unnecessarily complex
3. I think SEND is easy to use
4. I think that I would need the ongoing support of a technical person to be able to use SEND
5. I find that the various functions within SEND are well integrated
6. I think that there is too much inconsistency in the design of SEND
7. I would imagine that most people would learn to use SEND very quickly
8. I find SEND very cumbersome to use
9. I feel very confident using SEND
10. I needed to learn a lot of things before I could competently use SEND

Each question was rated by the respondent from strongly agree to strongly disagree. The system usability scale score was then calculated. To calculate the SUS score, the score contributions from each item was first summed. Each item's score contribution ranges from 0 to 4. For all odd-numbered items, the score contribution is the scale position (1 to 5) minus 1. For all even-numbered items, the contribution is 5 minus the scale position. The sum of the scores was multiplied by 2.5 to obtain a value out of 100.
